# Prevalence of subpatent *Plasmodium falciparum* infections in regions with varying transmission intensities and implications for malaria elimination in Mainland Tanzania

**DOI:** 10.1186/s12936-025-05341-6

**Published:** 2025-03-26

**Authors:** Misago D. Seth, Zachary R. Popkin-Hall, Rashid A. Madebe, Rule Budodo, Catherine Bakari, Beatus M. Lyimo, David Giesbrecht, Ramadhani Moshi, Ruth B. Mbwambo, Filbert Francis, Dativa Pereus, Doris Mbata, Daniel P. Challe, Salehe S. Mandai, Gervas A. Chacha, Angelina J. Kisambale, Daniel Mbwambo, Sijenunu Aaron, Abdallah Lusasi, Samwel Lazaro, Celine I. Mandara, Jeffrey A. Bailey, Jonathan J. Juliano, Julie R. Gutman, Deus S. Ishengoma

**Affiliations:** 1 National Institute for Medical Research, P. O. Box 9653, Dar es Salaam, Tanzania; 2 National Institute for Medical Research, Tanga, Tanzania; 3 University of North Carolina, Chapel Hill, NC USA; 4 Nelson Mandela African Institution of Science and Technology, Arusha, Tanzania; 5 The Connecticut Agricultural Experiment Station, New Haven, CT USA; 6 Muhimbili University of Health and Allied Sciences, Dar es Salaam, Tanzania; 7 National Malaria Control Programme, Dodoma, Tanzania; 8 Brown University, Providence, RI USA; 9 Centers for Disease Control and Prevention, Atlanta, GA USA; 10 Department of Biochemistry, Kampala International University in Tanzania, Dar es Salaam, Tanzania

**Keywords:** Subpatent infections, *Plasmodium falciparum*, Rapid diagnostic tests, qPCR, Malaria, Tanzania

## Abstract

**Background:**

Subpatent *Plasmodium falciparum* infections, defined as infections with parasite density below the detection limit of routine malaria diagnostic tests, contribute to infectious reservoirs, sustain transmission, and cause the failure of elimination strategies in target areas. This study assessed the prevalence of subpatent *P. falciparum* infections and associated risk factors in 14 regions of Mainland Tanzania.

**Methods:**

The study used samples randomly selected from RDT-negative dried blood spots (DBS) (n = 2685/10,101) collected in 2021 at 100 health facilities across 10 regions of Mainland Tanzania, and four communities in four additional regions. The regions were selected from four transmission strata; high (five regions), moderate (three regions), low (three regions), and very low (three regions). DNA was extracted by Tween-Chelex method, and the *Pf18S* rRNA gene was amplified by quantitative polymerase chain reaction (qPCR). Logistic regression analysis was used to assess the associations between age groups, sex, fever status, and transmission strata with subpatent infection status, while linear regression analysis was used to assess the association between these factors and subpatent parasite density.

**Results:**

Of the selected samples, 525/2685 (19.6%) were positive by qPCR for *P. falciparum*, and the positivity rates varied across different regions. Under-fives (aOR: 1.4, 95% CI 1.04–1.88; p < 0.05) from health facilities had higher odds of subpatent infections compared to other groups, while those from community surveys (aOR: 0.33, 95% CI 0.15–0.72; p = 0.005) had lower odds. Participants from very low transmission stratum had significantly lower odds of subpatent infection compared to those from high transmission stratum (aOR = 0.53, 95% CI = 0.37–0.78; p < 0.01). The log-transformed median parasite density (interquartile range) was 6.9 (5.8–8.5) parasites/µL, with significantly higher parasitaemia in the low transmission stratum compared to a very low one (11.4 vs 7.0 parasites/µL, p < 0.001).

**Conclusion:**

Even in very low transmission settings, the prevalence of subpatent infections was 13%, and in low transmission settings it was even higher at 29.4%, suggesting a substantial reservoir that is likely to perpetuate transmission but can be missed by routine malaria case management strategies. Thus, control and elimination programmes may benefit from adoption of more sensitive detection methods to ensure that a higher proportion of subpatent infections are detected.

**Supplementary Information:**

The online version contains supplementary material available at 10.1186/s12936-025-05341-6.

## Background

Malaria remains a significant public health concern in many parts of the world, particularly in sub-Saharan Africa (SSA) [1]. In 2022, over half of the 608,000 malaria deaths occurred in four SSA countries: Nigeria (31%), the Democratic Republic of the Congo (12%), Niger (6%), and the United Republic of Tanzania (4%) [1]. In Tanzania, malaria is a leading cause of morbidity and mortality, with 93% of the population living in areas where transmission occurs [2]. Malaria prevalence in Tanzania varies by region, and ranged from 0 to 23.4% in under-fives in 2022 [3]. Transmission intensity varies even within specific geographic areas [4–6]. This micro-epidemiology of malaria and the recent transition from holo/hyperendemic to hypoendemic transmission intensities need to be considered when planning for different malaria interventions, including those based on diagnostic and therapeutic methods.

Tanzania implements various strategies as core interventions to control and eventually eliminate malaria; including vector control, case management, and chemoprevention. The vector control interventions include insecticide-treated mosquito nets (ITNs), indoor residual spraying, and larval source management, while case management methods are based on prompt diagnosis using rapid diagnostic tests (RDTs) and effective treatment with artemisinin-based combination therapy [4, 7, 8]. Currently, the only chemoprevention method routinely used by the National Malaria Control Programme (NMCP) is intermittent preventive treatment in pregnancy using sulfadoxine-pyrimethamine (SP) [9]. Three more malaria preventive therapies including seasonal malaria chemoprevention (SMC), intermittent preventive treatment in infancy (IPTi) also known as perennial malaria chemoprevention (PMC), and intermittent preventive treatment in school children (IPTsc) have been adopted by NMCP [9], but only IPTsc has now been initiated in few selected councils in Mainland Tanzania.

Following the recent progress in reducing cases and deaths, Tanzania has set an ambitious target to eliminate malaria by 2030 as highlighted in the 2021–2025 National Malaria Strategic Plan (NMSP) [4]. In the current NMSP, enhanced surveillance, monitoring and evaluation, and response are highly prioritized to support the ongoing elimination efforts. In the current phase, which focuses on achieving malaria elimination, it is critical to ensure effective detection of malaria cases in very low transmission strata through passive, active, and reactive approaches. The efficacy of enhanced surveillance depends on the ability to detect as many infections as possible, including low-density subpatent infections, that are not detected by routine testing [10–12].

Subpatent *Plasmodium falciparum* infections account for a substantial proportion of malaria infections (nearly 50% in asymptomatic individuals), and thus create a challenge for malaria control and elimination efforts, as such infections contribute to ongoing transmission but often go undiagnosed and untreated [13, 14]. Due to the complex interaction between malaria immunity and transmission levels, no clear relationship between the prevalence of subpatent malaria infections and transmission intensity has been documented [13, 15–17]. Most studies conducted on the prevalence and significance of subpatent infections in areas with varying transmission intensities in Tanzania, have reported high prevalence in areas with low transmission intensities and low prevalence in areas with high transmission [18–24]. However, most of the studies report data that is limited in scope as each study covers a geographically distinct area, making it difficult to obtain a nation-wide picture.

Subpatent infections evade detection by routine diagnostic methods [13, 25, 26] and may act as reservoirs of and support ongoing transmission particularly in areas with very low transmission. Thus, in areas targeted for or implementing elimination strategies, this fraction of infection must be addressed with specific interventions. This study was conducted to determine the prevalence and risk factors associated with *P. falciparum* subpatent infections in individuals of all ages in 14 regions with varying malaria transmission intensity in Mainland Tanzania. The study provides the first nation-wide scan of subpatent infections and forms a platform for future studies of such infections, with a focus on regions closer to the elimination targets.

## Methods

### Study sites and population

**Fig. 1:**
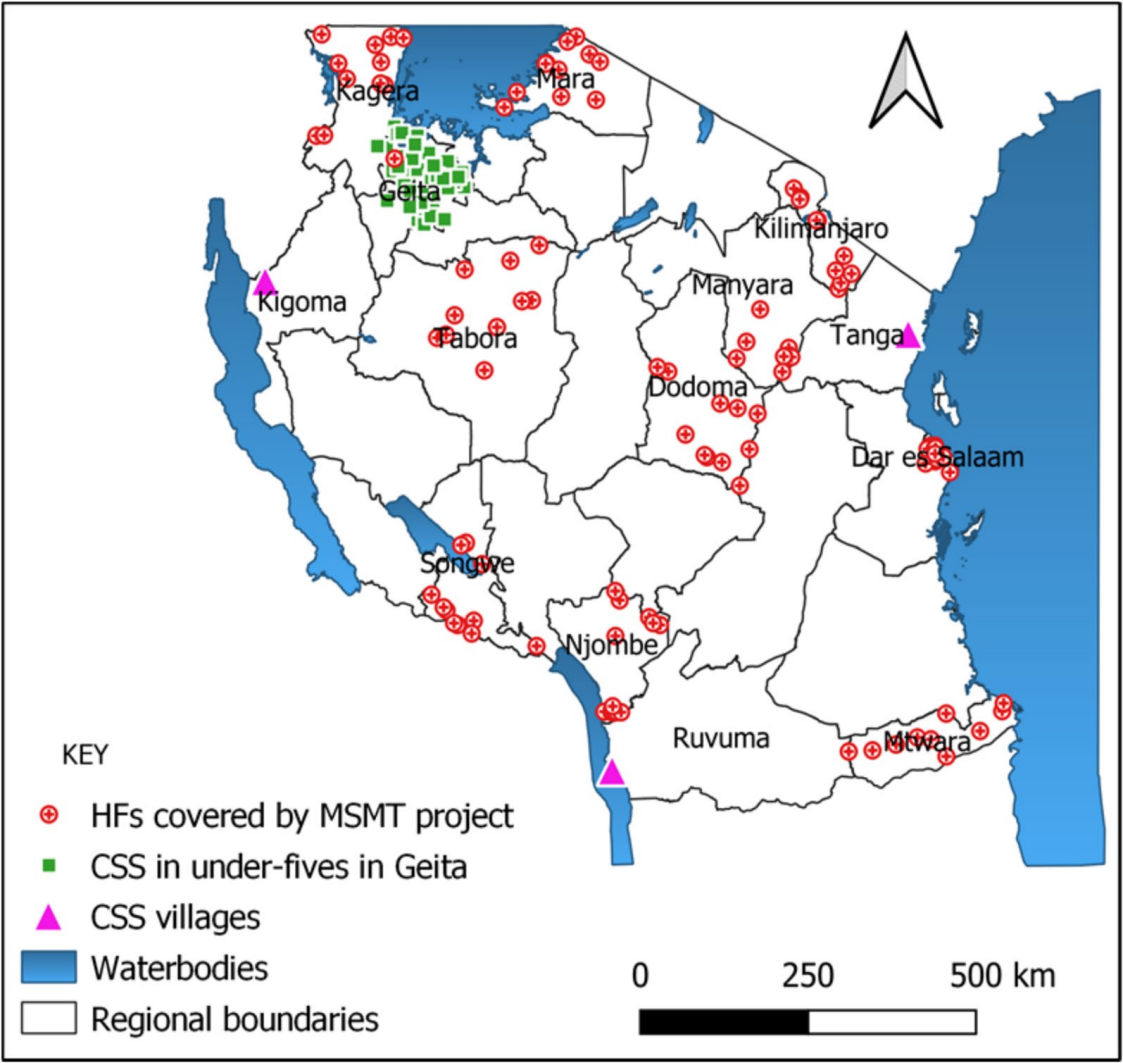
Map of Tanzania showing regions that were involved in the MSMT surveys in 2021. *HFs* health facilities, *CSS* community cross-sectional surveys

This study utilized data and samples collected from February to June 2021 in 14 regions as part of the project on molecular surveillance of malaria in Tanzania (MSMT) [27–29]. The study regions were selected among those located in four malaria burden strata based on the 2020 NMCP stratification; high (Geita, Kagera, Kigoma, Mtwara and Ruvuma), moderate (Mara, Tabora and Tanga), low (Dar es Salaam, Dodoma and Songwe) and very low (Kilimanjaro, Manyara and Njombe) (Fig. 1). In 10 regions (Dar es Salaam, Dodoma, Kagera, Kilimanjaro, Manyara, Mara, Mtwara, Njombe, Songwe and Tabora), cross-sectional health facility surveys were conducted in 10 health facilities per region, enrolling symptomatic patients with a history of fever in the preceding 48 h or fever at presentation (axillary temperature > 37.5 °C) [28, 29]. In four regions, participants were enrolled during community cross-sectional surveys (CSS) with samples collected irrespective of fever; in three of these (Kigoma, Ruvuma, and Tanga), individuals aged ≥ 6 months were enrolled, while in Geita, samples were collected from children aged 6–59 months only [29].

### Sample collection

**Fig. 2:**
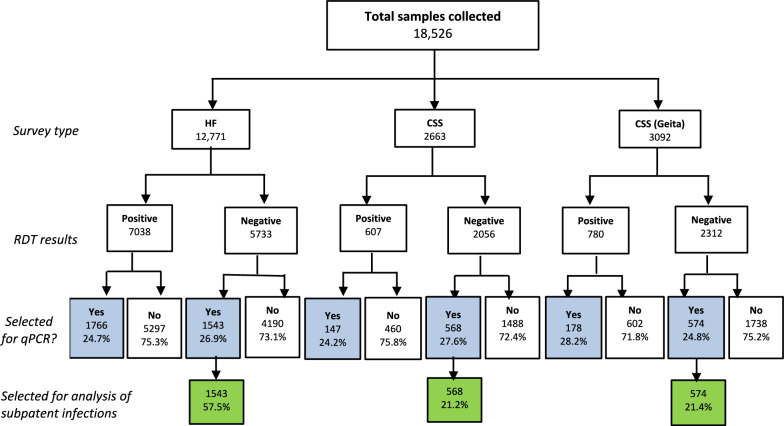
Flowchart showing how the samples used for this analysis were selected. *HF* health facility, *CSS* community cross-sectional survey

For each participant, finger prick blood was collected for detection of malaria infection by RDTs and preparation of dried blood spots (DBS) on Whatman 3 MM CHR filter papers (Cytiva, Marlborough, MA, USA) as described previously [28]. Different types of RDTs were used and they included SD Bioline Malaria Ag P.f/pan (#05FK60, Standard Diagnostic Inc., India), CareStart Malaria HRP2/pLDH (#RMOM-02571, AccessBio Inc., NJ, USA), and First Response Malaria Ag HRP2/pLDH Combo (#PI16FRC10s, Premier Medical Corp. India). A total of 18,526 DBS samples were collected in the 14 regions; 8425 (45.5%) were RDT positive and 10,101 (54.5%) were RDT negative. From these, 4776 (25.8%) were randomly selected for molecular analysis by quantitative polymerase chain reaction (qPCR) including 2685 (26.6%) RDT negative samples, which were analysed for subpatent *P. falciparum* infection (Fig. 2). The 4776 (25.8%) sub-sample selected for qPCR had over 100% power to detect differences in prevalence of subpatent infections based on the prevalences reported in previous studies [19, 21, 22]. Laboratory analysis of samples was performed at the National Institute for Medical Research (NIMR) Genomics Laboratory in Dar es Salaam, Tanzania.

### Laboratory analysis

#### DNA extraction

Genomic DNA was extracted from DBS samples (three punches of 6 mm each per sample) using Tween-Chelex 100 (Bio-Rad Laboratories, Hercules, CA, USA) as previously described, with minor modifications [28, 30]. Briefly, the punched DBS samples were incubated in 1 mL of 0.5% Tween-20 (Sigma) in phosphate-buffered saline (PBS) (Thermo Fisher Scientific, USA) and incubated on a shaker overnight at room temperature. After washing with 1 × PBS and boiling at 95 °C in Chelex 100 resin, a final volume of 150 μL of DNA was collected. After further centrifugation, OT-2 automated liquid handler (Opentrons Labworks, New York, USA) was used to make an aliquot of 50μL Chelex-free DNA. This aliquot was kept at − 20 °C until use in PCR assays.

#### Quantitative real-time PCR

Quantitative real-time PCR assay targeting the 18S ribosomal RNA (rRNA) was performed according to previously published methods [27, 29, 31]. Detection was done using TaqMan probe assay and parasitaemia quantification was based on standard curves generated using standard dilutions of plasmid DNA from MR4 (MRA-177, BEI Resources, Manassas, VA, USA) as previously described [27]. Parasitaemia was estimated based on the assumption of six 18S rRNA gene copies per parasite genome [31] and then multiplied by four to account for the dilution of eluted DNA relative to the initial blood volume [32].

### Data management and analysis

Data from community surveys were collected using tools configured and installed on tablets, running Open Data Kit (ODK) software. The data were directly transmitted to a central data server located at the National Institute for Medical Research (NIMR) in Dar es Salaam, Tanzania as described earlier [33, 34]. Health facility survey data were collected through paper questionnaires and double-entered into a Microsoft® Access® LTSC MSO (Version 2405) database. All the data were transferred to a Microsoft® Excel® LTSC MSO (Version 2405), cleaned, checked for consistency and transferred to Stata version 13 (STATA Corp, Inc., 2015) for further cleaning and analysis. Descriptive statistics, including means, frequencies, and proportions were used to summarize the data. Chi-square tests were used to assess bivariate relationships between categorical variables and the prevalence of subpatent infections. Univariate and multivariate logistic regression analyses were used to identify factors associated with subpatent malaria infection status. Relationships between variables were presented as odds ratios (ORs) with 95% confidence intervals (CIs). All the factors with p-values < 0.25 in the univariate analysis were included in the multivariate logistic regression model. Such variables were age group (under-fives, school children aged 5–15 and adults aged ≥ 15 years), sex, fever status and transmission strata. Multivariate Linear regression analysis was used to assess the association between parasite density and the independent variables such as age group, sex, fever status, and transmission stratum. The assumption of normality for the parasite density distribution was tested using histograms and Shapiro test. As the parasite density was not normally distributed, it was log-transformed using the natural logarithmic function and analysed to generate geometric mean parasite densities with 95% CI. The parasite densities in different strata were compared using Tukey’s honest significant difference (Tukey’s HSD) test. A p-value < 0.05 was considered statistically significant. The regional-level map of positivity rates of subpatent infections was created using the R package *sf* (version 1.0–9) based on shape files available from GADM.org and naturalearthdata.com accessed via the R package *rnaturalearth* (version 0.3.2).

## Results

### Characteristics of the study population

**A**mong 4776 (25.8%) samples for which qPCR results were available, 2685 (56.2%) were negative by RDT and were used for this analysis (Fig. 1b). For the participants who were included in the analysis, age and sex information were available for 2040 (76.0%) and 2046 (76.2%) participants, respectively. The median age (Interquartile range; IQR) of all participants was 8 years (4.4–25.7) and for the individuals selected for this study, the median age (IQR) was also 8 years (5–31) with the age ranging from 6 months to 87 years. Nearly half (46.0%, n = 1235/2685) of those included in this analysis were under-fives, 322 (12.0%) were school children (5–15 years), and 1128 (42.0%) were adults (> 15 years). The sex distribution was female-skewed, with 61.1% (n = 1251/2685) female participants, reflecting the gender distribution in the main dataset where 56.1% (n = 8651/18,526) were female (Table 1). The majority (57.5%, n = 1543/4776) of the participants of this study were drawn from health facility surveys which enrolled febrile/symptomatic patients (Fig. 2, Table 2).

**Table 1:** Demographic and clinical characteristics of individuals covered in the MSMT 2021 surveys and those selected for the analysis of subpatent infections

Variable	Total (N = 18,526)	Selected for qPCR n (%)	Pf subpatent infections n (%)		
			Total analysed	With Pf Subpatent infection	
Total	18,526	4776	2685	525	
Age group^a^					
< 5 years	8006	1919 (40.2)	1235	282 (22.8)	
5–15 years	3330	971 (20.3)	322	58 (18.0)	
15 + years	7190	1886 (39.5)	1128	185 (16.4)	
Sex^b^					
Female	8651	2282 (58.1)	1251	208 (16.6)	
Male	6782	1643 (48.9)	795	125 (15.7)	
Fever^c^					
At presentation	7614	1509 (38.8)	658	109 (16.6)	
History of fever (48 h)	5692	1868 (48.0)	926	150 (16.2)	
No fever	1962	516 (13.3)	445	81 (18.2)	
Transmission strata					
High	7412	1931 (40.4)	1,161	242 (20.8)	
Moderate	3492	867 (18.2)	361	89/ (24.7)	
Low	2585	640 (13.4)	262	77 (29.4)	
Very low	5037	1338 (28.0)	901	117 (13.0)	
Regions					
Dar es Salaam	753	214 (4.5)	62	16 (25.8)	
Dodoma	1005	279 (5.8)	149	58 (38.9)	
Kagera	1515	419 (8.8)	159	35 (22.0)	
Kilimanjaro	3331	897 (18.8)	736	94 (12.8)	
Manyara	852	229 (4.8)	81	9 (11.1)	
Mara	1357	337 (7.1)	101	24 (23.8)	
Mtwara	1179	304 (6.4)	61	17 (27.9)	
Njombe	854	212 (4.4)	84	14 (16.7)	
Songwe	827	147 (3.1)	51	3 (5.9)	
Tabora	1098	271 (5.7)	59	17 (28.8)	
Kigoma	883	253 (5.3)	207	10 (4.8)	
Ruvuma	743	203 (4.3	160	39 (24.4)	
Tanga	1037	259 (5.4)	201	48 (23.9)	
Geita	3092	752 (15.7)	574	141 (24.6)	

^a,b,c^Age, sex, and fever information was not available for all participants

Pf = *Plasmodium falciparum*; *qPCR* quantitative Polymerase Chain Reaction

**Table 2:** Demographic and clinical characteristics of individuals selected for analysis of *P. falciparum* subpatent infections from cross-sectional health facility surveys (in10 regions) and community surveys (in four regions) in Mainland Tanzania

Variable	HF Survey		CSS^a^	
	Total analysed	Pf Subpatent infections	Total analysed	With Pf Subpatent infections
Total	1,543	287 (18.6)	568	97 (17.1)
Age group^b^				
< 5 years	560 (36.3)	133 (23.8)	79 (13.9)	8 (7.9)
5–15 years	196 (12.7)	39 (19.9)	142 (25.0)	19 (15.1)
15 + years	787 (51.0)	115 (14.6)	347 (61.1)	70 (20.5)
*p-value*	*1*	< *0.001*		*0.002*
Sex^c^				
Female	841 (56.7)	136 (16.2)	410 (72.8)	72 (17.5)
Male	642 (43.3)	103 (16.0)	153 (27.2)	22 (14.4)
*p-value*		*0.947*		*0.86*
Fever history (past 48 h)^d^				
No	132 (9.0)	26 (19.7)	344 (61.1)	53 (15.4)
Yes	1342 (91.0)	211 (15.7)	219 (38.9)	41 (18.7)
*p-value*		*0.236*		*0.332*
Fever at presentation (a. temp. ≥ 37.5 °C)^d^				
No	825 (56.0)	129 (15.6)	550 (96.8)	94 (17.1)
Yes	649 (44.0)	108 (16.6)	18 (3.2)	0 (0.00)
*p-value*		*0.602*		*0.84*
Transmission strata				
High	220 (14.3)	52 (23.6)	367 (64.6)	57 (15.5)
Moderate	160 (10.4)	41 (25.6)	201 (35.4)	48 (23.9)
Low	262 (17.0)	77 (29.4)	NA	NA
Very low	901 (58.4)	117 (13.0)	NA	NA
*p-value*		< *0.001*		*0.014*
Regions				
Dar es Salaam	62 (4.0)	16 (25.8)		
Dodoma	149 (10.0)	58 (38.9)		
Kagera	159 (10.3)	35 (22.0)		
Kilimanjaro	736 (47.7)	94 (12.8)		
Manyara	81 (5.2)	9 (11.1)		
Mara	101 (6.5	24 (23.8)		
Mtwara	61 (4.0)	17 (27.9)		
Njombe	84 (5.4)	14 (16.7)		
Songwe	51 (3.3)	3 (5.9)		
Tabora	59 (3.8)	17 (28.8)		
Kigoma			207 (36.4)	18 (8.7)
Ruvuma			160 (28.2)	39 (24.4)
Tanga			201 (35.4)	48 (23.9)
Geita^e^			574 (21.4)	141 (24.6)
*p-value*		< *0.001*		< *0.001*

^a^The CSS group involves communities from high and moderate strata only because the survey was conducted conveniently and utilized an existing platform of communities for which census data is available

^b,c,d^ Age, sex, and fever information was not available for all participants

^e^Sampling in Geita involved only under-fives and most clinical and demographic parameters were not collected. Therefore, samples from Geita are not presented with the rest of the CSS regions in the above table. The total samples analysed in the CSS group (n = 568), and those with subpatent infections (n = 97) does not include Geita samples

Pf = *Plasmodium falciparum;* a. temp. = axillary temperature

### Prevalence of subpatent infections

Among the 2685 RDT-negative samples tested, 525 (19.6%) were positive by qPCR (Table 1).

#### Health facility survey

In the health facility survey, the overall prevalence of subpatent infections was 18.6% (287/1543); and it was significantly higher in under-fives (23.8%, n = 133/560) compared to other age groups (p < 0.001). There were no significant differences by sex in the prevalence of subpatent infections (16.2% in females vs 16.0% in males, unadjusted OR: 1.01, 95% CI 0.76–1.34; p = 0.947). The prevalence of sub-patent infection was also similar in individuals with or without fever at presentation or history of fever within 48 h before the survey (p > 0.237 for both comparisons) (Table 2). School children had slightly, but not statistically significantly, higher odds of subpatent infections than children under-five children (aOR: 1.24, 95% CI 0.18–1.88; p = 0.307).

The prevalence was significantly lower in the very low transmission stratum (13.0%) compared to high transmission strata (23.6%; aOR: 0.53, 95% CI 0.37–0.78; p < 0.001). The prevalence of subpatent infection was similar in low, moderate and high transmission strata (p > 0.05 for all comparisons). The prevalence of subpatent infections was particularly high in Dodoma region (38.9%), making the overall prevalence highest in the low transmission stratum;  (29.4%). Sensitivity analysis excluding Dodoma region demonstrated that the prevalence in low transmission areas dropped significantly to 16.8%. This was lower than the reported prevalence in high and moderate transmission areas (aOR: 0.65, 95% CI 0.36–1.16, p = 0.146).

#### Cross-sectional survey

**Fig. 3:**
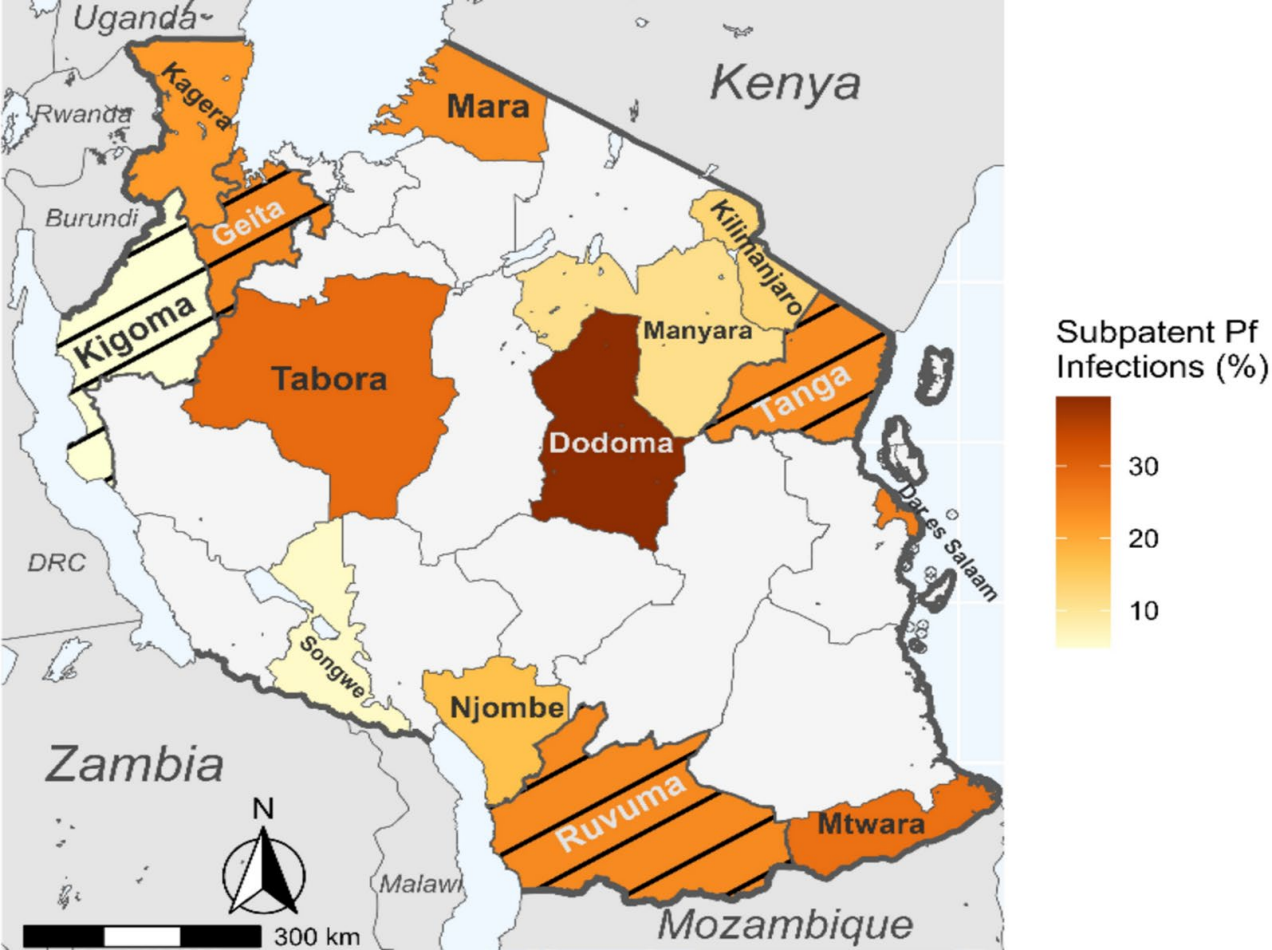
Map of Tanzania showing the proportion of symptomatic patients or asymptomatic individuals with subpatent infections in the studied regions. Black bars indicate regions that were involved in CSS surveys (Geita, Kigoma, Ruvuma, and Tanga), while the regions with solid fill were involved in health facility surveys

In the CSS group that involved the three regions of Kigoma, Ruvuma and Tanga, the prevalence of subpatent infections was 17.1% (n = 97/568) and was significantly lower in under-fives (7.9%) compared to older individuals (> 15.0%; p = 0.002), and in individuals from high compared to moderate transmission stratum (23.9 vs 15.5%; p = 0.01). The prevalence was similar among individuals of different sex (females 17.5% and males 14.4%; unadjusted OR: 1.27, 95% CI 0.76–2.13; p = 0.369) and those with or without fever at presentation or history of fever within 48 h before the survey (18.7% vs 15.4%; OR 1.26, 95% CI 0.81–1.98; p = 0.31) (Tables 2, 3). After adjusting for sex, transmission strata, fever status, and survey type, the risk of subpatent parasitaemia remained significantly lower in under-fives compared to older individuals (aOR: 0.33, 95% CI 0.15–0.72, p = 0.005) (Table 3). The prevalence of subpatent infections was 24.6% (141/574) among under-fives in Geita region; significantly higher compared to that of under-fives in the other three CSS regions (7.9%; p < 0.001) (Fig. 3).

**Table 3:** Logistic regression analysis to determine the odds of subpatent infections among RDT-negative samples from 14 regions of Mainland Tanzania

Characteristics	Health facilities survey		Community survey	
	uOR, 95% CI, p-value	aOR, 95% CI, p-value	uOR, 95% CI, p-value	aOR, 95% CI, p-value
Transmission strata				
High	Reference	Reference	Reference	Reference
Moderate	1.11 (0.69–1.78), 0.66	1.10 (0.69–1.77), 0.69	2.04 (1.31- 3.17), 0.002	2.05 (1.31–3.21), 0.002
Low	1.34 (0.89–2.02), 0.16	1.35 (0.89–2.04), 0.148		
Very low	0.48 (0.33–0.70), < 0.001	0.53 (0.37–0.78), < 0.001		
Sex				
Male	Reference		Reference	
Female	1.01 (0.76- 1.34), 0.95		1.27 (0.76–2.13), 0.37	
History of fever (past 48 h) group^b^				
No	Reference		Reference	
Yes	0.76 (0.48- 1.20), 0.24		1.26 (0.81–1.98), 0.31	
Fever at presentation (atemp ≥ 37.5 °C)				
No	Reference			
Yes	1.08 (0.81- 1.42), 0.60			
Age group				
< 5 years	1.82 (1.38–2.4), < 0.001	1.40 (1.04–1.88),0.03	0.33 (0.15–0.72),0.005	0.33 (0.15–0.72),0.005
5–14 years	1.45 (0.97–2.17), 0.07	1.29 (0.85–1.94), 0.23	0.69 (0.39–1.20), 0.19	0.67 (0.38–1.18), 0.16
15 + years	Reference	Reference	Reference	Reference

^a,b^During health facility surveys, recruited patients either had history of fever or measured figure at point of enrolment, such that patients that did not present with fever, were enrolled due to their history of fever in the past 48 h

*atemp* axillary temperature, *aOR* adjusted odds ratio, *uOR* unadjusted odds ratio

### Parasite density

**Fig. 4:**
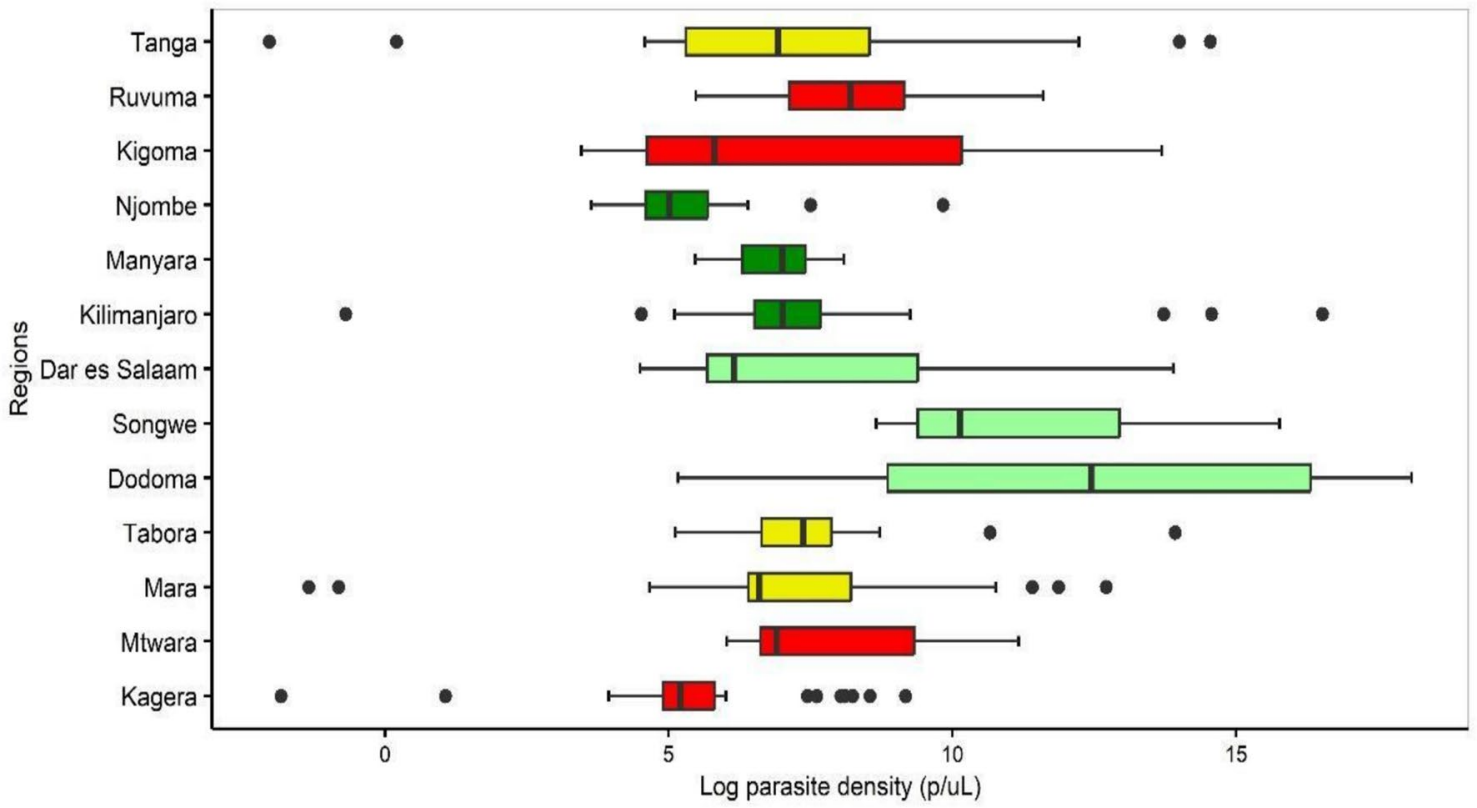
Median parasite densities in the different regions across transmission intensities. Transmission strata are classified as high (red), moderate (yellow), low (light green), and very low (green)

**Fig. 5:**
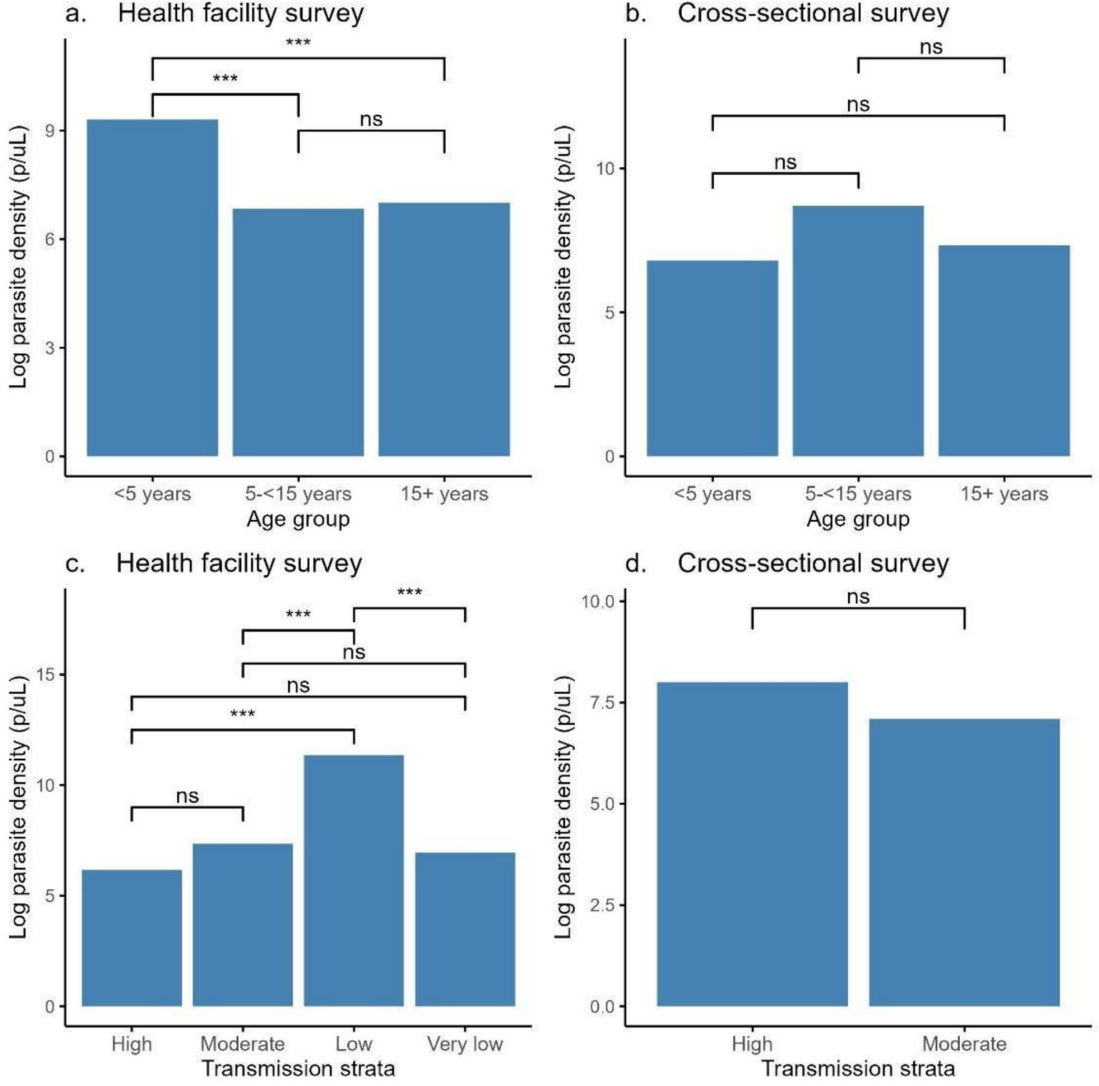
Tukey analysis of subpatent parasite density by age groups (a[health facility survey]; b[community survey]) and transmission strata (c[health facility survey]; d[community survey]). Level of significance is shown as follows: **p < 0.01, ***p < 0.001, *ns* no significant difference

Parasite densities (geometric mean of asexual parasites per microlitre of blood (p/µL)) for subpatent infections varied significantly among the regions (p < 0.001). The log-transformed median parasite density (IQR) was 6.9 (5.8–8.5) p/µL. Overall, parasite density was significantly higher in the low compared to very low transmission stratum (11.4 vs 7.0 p/µL, p < 0.001). The highest parasite density was in samples from Dodoma region (log_10_ 12.4 (8.8–16.3) p/µL) and the lowest in Njombe (5.0, IQR = 4.5–5.7p/µL) (Fig. 4). Linear regression analysis revealed similar parasite densities among males and females in the health facility survey samples (males had 0.51 p/µL less than females, 95% CI −1.13–0.11, p = 0.11) while among those sampled in CSS, males (8.62 p/µL) had a statistically higher average parasite density than females (7.18 p/µL) (Adj Coef β 1.38 p/µL (95% CI 0.06–2.70, p = 0.04), though from a clinical standpoint this is not different (Table 4). In the CSS, the parasite density increased in school children (aged 5–15 years) compared to adults aged > 15 years (8.7 p/µL vs 7.33 p/µL; Adj. Coef β 1.42, 95% CI 0.13–2.70, p = 0.03), and increased non-significantly in under five children from health facility surveys (Table 4). Tukey analysis of CSS parasitaemia data revealed higher but non-significant mean parasite density in school children, and individuals from high (8.0 p/µL) as compared to those from regions located in moderate (7.1 p/µL) transmission strata, while in the health facility surveys, the mean parasite density was significantly higher in under-fives (8.1 p/µL) than in older age groups (6.1 p/µL for school children and 7.0 p/µL for adults), and in individuals from low (11.4 p/µL) versus high (6.7 p/µL) transmission strata (Fig. 5).

**Table 4:** Linear regression analysis of factors associated with parasite density of subpatent infections among RDT-negative samples from 14 regions of Mainland Tanzania

	Health facility model				Cross-sectional survey model			
Variable	Crude Coef β	95% CI, P-value	Adj Coef β	95% CI, P-value	Crude Coef β	95% CI, P-value	Adj Coef β	95% CI, P-value
Sex								
Male	− 0.431	− 1.052–0.191, p = 0.173	− 0.51	− 1.128–0.109, p = 0.106	1.437	0.2 00–2.674, p = 0.023	1.382	0.061–2.703, p = 0.041*
Female		Reference		Reference		Reference		Reference
Age in years	− 0.009	− 0.025–0.007, p = 0.284			− 0.025	− 0.049–0.002, p = 0.04		
Age group								
< 5 years	2.297	1.464–3.131, p = 0.003	0.194	− 0.494–0.882, p = 0.579	− 0.533	− 2.452–1.386, p = 0.583	− 1.54	− 3.859–0.78, p = 0.191
5–15 years	− 0.172	− 1.385–1.041, p = 0.78	− 0.269	− 1.153–0.616, p = 0.55	1.374	0.043–2.704, p = 0.043	1.416	0.133–2.7, p = 0.031*
> 15 years		Reference		Reference		Reference		Reference
Transmission strata								
Moderate	1.189	0.005–2.373, p = 0.049	1.248	0.251–2.245, p = 0.014	− 0.905	− 1.954–0.145, p = 0.09	− 0.162	− 1.292–0.967, p = 0.776
Low	5.187	4.169–6.205, p = 0	2.276	1.181–3.372, p < 0.001				
Very low	0.789	− 0.156–1.734, p = 0.102	0.759	− 0.039–1.558, p = 0.062				
High		Reference		Reference				
Fever at presentation								
Yes	− 0.157	− 0.781–0.470, p = 0.622	− 0.378	− 0.999–0.244, p = 0.232				
No		Reference		Reference				

*atemp* axillary temperature, *aOR* adjusted odds ratio, *uOR* unadjusted odds ratio

## Discussion

Subpatent malaria infections are potential reservoirs for persistent malaria transmission, thus, they are a threat to the ongoing malaria elimination plans in malaria-endemic countries [13, 15], particularly in elimination or pre-elimination areas [13, 25, 35–38]. Due to this threat, areas nearing elimination programmes must ensure that all cases, including subpatent infections, are tracked and treated. Particularly in low transmission areas, where the prevalence of subpatent infections was the highest, this requires more sensitive diagnostic tools [35, 39, 40].

The present study demonstrated that the prevalence of subpatent *P. falciparum* infections is high, at 19.6% overall, with marked heterogeneity among the studied regions. The positivity rates were higher in regions with low transmission (similar to moderate and high transmission areas) and then fell in those with very low transmission. The heterogeneity in subpatent infections reflects an inverse relationship to the patent malaria infections which has been reported in areas of different endemicity in Mainland Tanzania [4–6] and elsewhere [15, 25, 41]. The heterogeneity in patent infections is attributed to scaled-up interventions, which have resulted in a shift of malaria epidemiology, with some areas transitioning from hyper-endemic to hypo-endemic transmission [4, 42]. The high prevalence of subpatent infections in areas of low transmission highlights the urgent need to design and implement more efforts to eliminate malaria. The prevalence of subpatent infections was particularly high in Dodoma region and the reasons for such prevalence are not clearly known. Sensitivity analysis excluding Dodoma suggested that Dodoma region may be driving the high likelihood of subpatent infections in low transmission areas. When Dodoma was excluded, there was a low likelihood of subpatent infections in low compared to high and moderate transmission areas. Thus, future studies are needed to monitor these trends and confirm the pattern seen in this study.

Subpatent infections were less prevalent in asymptomatic under-fives than in older age groups. This is most likely because this group has had the least exposure to malaria and thus have not yet developed naturally acquired immunity to suppress the infection, so most infections are patent and cause clinical symptoms [43]. Many studies have reported a higher prevalence of subpatent infections in adults than in children, as well as in older children compared to younger children [13, 16, 18, 25]. School children exhibit a high prevalence of patent infections, most of which are asymptomatic due to developing immunity [33, 34]. Adults are more likely to have subpatent infections, as increasing age is associated with an increase in naturally acquired immunity, which suppresses the parasites to low densities that may be undetectable by routine tests [43]. The high odds of *P. falciparum* subpatent infections in symptomatic under five children could be due to residual parasitaemia following treatment with anti-malarials or detection of DNA of persisting gametocytes as previously described although no analysis was done to confirm this [37, 44, 45]. However, this may not be in line with the findings from other settings where children had the lowest prevalence [23] or other studies that did not find any association between age groups and subpatent infections [46]. Variations in the prevalence of subpatent infections among different age groups from different settings warrants further exploration.

This study did not find any significant association between fever and the prevalence of subpatent infections in participants recruited from health facilities, underscoring that these infections often remain asymptomatic, as has been reported from Malawi [47, 48]. The lack of a significant association could also be related to the fact that the majority of individuals recruited from health facilities had a history of fever or had fever at the time of sample collection. In contrast, in community surveys, where most people were asymptomatic, submicroscopic parasitaemia was associated with fever. This was also reported in a cohort study in Uganda [49], indicating that subpatent infections may cause fever, although it is also possible that the fever was due to other concomitant infections. More studies to explore the contribution of subpatent malaria infection to fever are recommended.

The prevalence of subpatent infections increased with decreasing transmission intensity, but dropped again as transmission intensity became very low. This finding is in line with what has been reported previously [15, 25], although other studies have reported a low prevalence of subpatent infections in low-transmission settings [50]. One possible reason could be that in high-transmission areas, the greater exposure to infectious bites results in more people with higher density infections compared to low transmission areas. These are then more likely to be detected and treated, clearing the infection [36].

In this study, the lowest prevalence of subpatent infections was found in very low transmission areas, such as Kilimanjaro, Manyara, and Njombe regions. In these areas, most people have likely lost any naturally acquired immunity to malaria they might have had; thus, the majority of infections become symptomatic and are treated [43]. In order to achieve the elimination goals stated in the 2021–2025 strategic plan, which proposes transitioning to malaria elimination in phases, the National Malaria Control Programme now implements malaria case-based surveillance pilots in areas with very low malaria burden, starting with three northern regions of Arusha, Kilimanjaro, and Manyara, with a plan to scale up to other very low malaria burden regions [4]. While rates of subpatent infections were lowest in these areas, subpatent infections were still detected in 13.0% of the tested individuals, which is high compared to what was reported in pre- and elimination areas in other countries where subpatent infection rates ranged between zero and five percent [17, 38, 49, 51–53]. Apart from gauging transition criteria based on patent infection prevalence and incidence, it may be necessary to also consider approaches targeting subpatent infections with the aim of reducing them to a level that does not pose a transmission threat in elimination settings. Therefore, more sensitive tests should be considered to improve the detection of subpatent infections.

The parasite densities were generally low and heterogeneous across different regions and transmission strata. The highest average parasite density was found in the low transmission strata. This supports findings of other studies reporting that subpatent parasite density was negatively associated with transmission intensity [18, 54, 55]. The high parasite density in young children could be explained by their less developed immunity and could also indicate parasites transitioning from subpatent to patent status over time. Younger children are at higher risk of malaria infection, and the high density of subpatent infections, even in areas of low transmission, calls for continued attention to this group. There was a negative association between parasite density and fever, with febrile individuals having low-density subpatent infections. This is partially attributed to the fact that patients with patent parasitaemia were not included in the modeling, and likely reflects that these individuals had an alternative etiology for their fever. Nonetheless, in the context of malaria control and elimination, even low-density infections can be infectious to mosquitoes, maintaining transmission in the population, and are thus important to identify and treat [13, 38, 56]. While majority of subpatent infections are due to low density parasitaemia; in situations where HRP2-based RDTs are used, deletion of the histidine-rich protein 2 and histidine-rich protein 3 genes (*pfhrp2* and *pfhrp3*) has also been implicated as a reason for the failure of RDTs to detect *P. falciparum* even with higher density infections [57–59]. It is important to continue monitoring for the occurrence and spread of hrp2/3 gene deleted parasites.

This study was part of a main study designed to maximize collection of RDT-positive samples. This may have affected representativeness of transmission strata since even health facilities from low transmission may have come from areas with relatively higher transmission compared to the rest of the region. Future studies should consider random selection of health facilities to address this selection bias.

## Conclusion

Subpatent infections are common and heterogeneous in most parts of mainland Tanzania. Even in very low and low transmission settings, the prevalence of subpatent infections was still high, suggesting a substantial reservoir which is likely to be missed by routine malaria case management strategies. To manage subpatent infectious reservoirs in regions with very low levels of *P. falciparum* transmission, elimination programmes may consider adopting more sensitive detection methods for the case-based management (test-and-treat) approach or mass drug administration that does not rely on the sensitivity of diagnostic tests.

## Supplementary Information

Supplementary Material 1

## Data Availability

No datasets were generated or analysed during the current study.
