# Supplementary material for: Prevalence of subpatent Plasmodium falciparum infections in regions with varying transmission intensities and implications for malaria elimination in Mainland Tanzania
Source: Malar J. 2025 Mar 26;24:101. doi: 10.1186/s12936-025-05341-6 (PMC11948789; doi:10.1186/s12936-025-05341-6)
Supplement: Supplementary file 1 — Supplementary Material 1 [file 12936_2025_5341_MOESM1_ESM.docx]

**Supplementary Table S1**

| **Variable** | **Population** | **Sample** | **χ2, p-value** |
| --- | --- | --- | --- |
| **Total** | 18,526 | 4,776(25.8) | 20.1, p< 0.001 |
| **Age group^a^** |  |  |  |
| <5 years | 8006(43.2) | 1,919(40.2) |  |
| 5-15 years | 3330(18.0) | 971(20.3) |  |
| 15+years | 7190(38.8) | 1,886(39.5) |  |
| **Sex^b^** |  |  | 5.5, p=0.019 |
| Female | 8,651(56.1) | 2,282(58.1) |  |
| Male | 6,782(43.9) | 1,643(41.9) |  |
| **Fever history, (past 48 hours**) |  |  | 0.01, p = 0.912 |
| No | 2,487 (16.2) | 636(16.3) |  |
| Yes | 12,848 (83.8) | 3,268(83.7) |  |
| **Fever at presentation (atemp≥37.5°C)** |  |  | 1.0, p = 0.309 |
| No | 7,695(50.1) | 2,032(49.2) |  |
| Yes | 7,659(49.9) | 2,096(50.8) |  |
| **Transmission strata** |  |  | 2.9, p= 0.401 |
| High | 7,412(40.0) | 1931(40.4) |  |
| Moderate | 3,492(18.8) | 867(18.2) |  |
| Low | 2,585(14.0) | 640(13.4) |  |
| Very low | 5,037(27.2) | 1338(28.0) |  |
| **Regions** |  |  | 29.5, p = 0.005 |
| Dar es Salaam | 753(4.1) | 214(4.5) |  |
| Dodoma | 1,005(5.4) | 279(5.8) |  |
| Kagera | 1515(8.2) | 419(8.8) |  |
| Kilimanjaro | 3,331(18.0) | 897(18.8) |  |
| Manyara | 852(4.6) | 229(4.8) |  |
| Mara | 1,357(7.3) | 337(7.1) |  |
| Mtwara | 1,179(6.4) | 304(6.4) |  |
| Njombe | 854(4.6) | 212(4.4) |  |
| Songwe | 827(4.5) | 147(3.1) |  |
| Tabora | 1,098(5.9) | 271(5.7) |  |
| Kigoma | 883(4.8) | 253(5.3) |  |
| Ruvuma | 743(4.0) | 203(4.3 |  |
| Tanga | 1037(5.6) | 259(5.4) |  |
| Geita | 3,092(16.7) | 752(15.7) |  |
